# A coordinate-based meta-analysis of white matter alterations in patients with alcohol use disorder

**DOI:** 10.1038/s41398-022-01809-0

**Published:** 2022-01-27

**Authors:** Carolin Spindler, Louisa Mallien, Sebastian Trautmann, Nina Alexander, Markus Muehlhan

**Affiliations:** 1 Department of Psychology, Faculty of Human Sciences, Medical School Hamburg, Am Kaiserkai 1, 20457 Hamburg, Germany; 2 ICAN Institute for Cognitive and Affective Neuroscience, Medical School Hamburg, Am Kaiserkai 1, 20457 Hamburg, Germany; 3 Department of Human Medicine, Faculty of Medicine, Medical School Hamburg, Am Kaiserkai 1, 20457 Hamburg, Germany; 4 ICPP Institute for Clinical Psychology and Psychotherapy, Medical School Hamburg, Am Kaiserkai 1, 20457 Hamburg, Germany; 5 Department of Psychiatry and Psychotherapy, Philipps University Marburg, Rudolf-Bultmann-Str. 8, 35039 Marburg, Germany; 6 Center for Mind, Brain and Behavior, Philipps University Marburg, Hans-Meerwein-Str. 6, 35032 Marburg, Germany

**Keywords:** Pharmacology, Addiction, Neuroscience

## Abstract

*Introduction:* Besides the commonly described gray matter (GM) deficits, there is growing evidence of significant white matter (WM) alterations in patients with alcohol use disorder (AUD). WM changes can be assessed using volumetric and diffusive magnetic resonance imaging methods, such as voxel-based morphometry (VBM) and diffusion tensor imaging (DTI). The aim of the present meta-analysis is to investigate the spatial convergence of the reported findings on WM alterations in AUD. *Methods:* Systematic literature search on PubMed and further databases revealed 18 studies eligible for inclusion, entailing a total of 462 AUD patients and 416 healthy controls (up to January 18, 2021). All studies that had used either VBM or DTI whole-brain analyzing methods and reported results as peak-coordinates in standard reference space were considered for inclusion. We excluded studies using approaches non-concordant with recent guidelines for neuroimaging meta-analyses and studies investigating patient groups with Korsakoff syndrome or other comorbid substance use disorders (except tobacco). *Results:* Anatomical likelihood estimation (ALE) revealed four significant clusters of convergent macro- and microstructural WM alterations in AUD patients that were assigned to the genu and body of the corpus callosum, anterior and posterior cingulum, fornix, and the right posterior limb of the internal capsule. *Discussion:* The changes in WM could to some extent explain the deteriorations in motor, cognitive, affective, and perceptual functions seen in AUD. Future studies are needed to clarify how WM alterations vary over the course of the disorder and to what extent they are reversible with prolonged abstinence.

## Introduction

Alcohol use disorder (AUD) is highly prevalent worldwide, leads to extensive health and economic burdens, and represents a leading cause of preventable deaths [1, 2].

Heavy chronic alcohol consumption causes numerous somatic diseases (e.g., liver disease and cancer) [3, 4] and poses damage to the central nervous system (CNS) [5, 6]. As a consequence, impaired cognitive functioning and, in severe cases, alcohol-related dementia have been frequently described in AUD patients [7]. Several processes might account for neurodegeneration in AUD, such as the toxic effects of ethanol and its metabolites itself as well as the frequent co-existing nutritional or vitamin deficiencies [8]. Moreover, dysregulations of central stress response systems due to chronic alcohol consumption and inflammatory mechanisms are also discussed as potential causes of CNS damages in AUD [9–11].

Specifically, these multiple factors can lead to injuries of neurons as well as glial cells of all types and induce demyelination and axonal damage, depending on dose and duration of exposure [9, 12]. Furthermore, age, sex as well as comorbid neurological and psychological conditions are discussed as potential moderators in this context [7, 13]. Intriguingly, some of these structural damages might be partially reversible with prolonged abstinence ([14], see refs. [7–11, 15] for review).

Structural brain alterations in AUD have been investigated in a large number of neuroimaging studies. Regarding GM changes, several meta-analyses highlighted specific patterns of reduced regional brain volume or density [16–18]. In recent years, the number of studies investigating the macro- and microstructure of WM by magnetic resonance imaging (MRI) methods has also increased. These studies showed WM changes in numerous locations of different fiber tracts (e.g., [19–22] and [23, 24] for review).

A first meta-analysis by Monnig and colleagues [25] revealed significant WM volumetric reductions in AUD relative to healthy comparison groups. This important work showed small to moderate effect sizes (*g* = 0.304, SD = 0.134) but did not test for convergence of imaging results in order to draw conclusions about the location of altered WM in AUD. A recent large-scale meta-analysis focused on gray and white matter morphology across all substance use disorders and indicates general and substance-specific structural brain changes in patients compared with healthy controls [26]. Sub-analysis regarding AUD in specific revealed convergent WM alterations in regions of the corticospinal tract and anterior thalamic radiation. However, these findings refer to the macrostructural results of only five voxel-based morphometry (VBM) studies and should be interpreted with great caution due to the low statistical power [27].

To date, a coordinate-based meta-analysis specifically focusing on the localization of WM alterations in AUD with sufficient power to generate robust results is still lacking [27, 28]. Our work aims to fill this gap by combining volumetric and diffusion-based MRI results of prior studies.

Besides the voxel-by-voxel volumetric comparisons, as it is done in VBM methods [29, 30], diffusion tensor imaging (DTI) can be used to determine the directionality of the diffusion of hydrogen protons, which is assumed to provide information about the microstructural integrity of the fiber tracts [31, 32]. Although volumetric and diffusion-based methods measure different features of WM, both provide information on WM changes in patient groups compared to healthy controls, and meta-analytically integrating results of studies applying one of both methods can give a more comprehensive overview of the affected brain regions (e.g., [33]).

To identify VBM and DTI studies on WM macro- and microstructural changes in AUD, we first conducted a systematic literature review in accordance with the Preferred Reporting Items for Systematic reviews and Meta-Analyses (PRISMA) [34]. The reported spatial coordinates of each study were extracted, weighted, and tested for spatial convergence using anatomical likelihood estimation (ALE). This method allows quantitative and unbiased integration of neuroimaging findings. Current guidelines for quantitative coordinate-based meta-analyses [27, 28] were meticulously followed in all steps of the work.

## Methods

Details of the protocol for this meta-analysis were registered on PROSPERO and can be assessed at https://www.crd.york.ac.uk/prospero/display_record.php?ID = CRD42021231447/display_record.php?ID = CRD42021231447.

### Literature search, study selection, and data extraction

The search for neuroimaging studies investigating WM alterations in patients with AUD compared to healthy controls was conducted up to January 18, 2021, on PubMed and on EBSCO hosted PsycINFO, PsycARTICLES, MEDLINE Complete, CINAHL Complete, and Psychology and Behavioral Sciences Collection databases were used as well as reference-tracing of the retrieved articles. Database filters were set for: Humans, English, Peer-Reviewed.

Keywords were: (alcohol misuse OR alcoholism OR alcohol drinking OR drinking behavior OR binge drinking OR alcoholics OR alcohol use disorder OR alcohol dependence OR alcohol addiction OR chronic alcoholic intoxication OR alcohol abuse) AND (white matter OR white brain matter OR cerebellar white matter OR white matter integrity) AND (diffusion tensor* OR DTI OR magnetic resonance imaging OR tractography OR mean diffusivity OR axial diffusivity OR radial diffusivity OR fractional anisotropy OR structural connectivity OR structural changes OR structural MRI OR voxel-based morphometry OR VBM).

Study inclusion criteria comprised (1) written in English language and peer-reviewed, (2) contains a statistical comparison of WM by means of VBM or DTI in the whole brain, (3) compares adult patients diagnosed with AUD (DSM-IV, DSM-5, or ICD-10) with healthy controls. AUD (as specified in DSM-5) is referred to as a disorder continuum subsuming DSM-IV diagnosis of alcohol abuse and alcohol dependence as well as ICD-10 diagnosis of harmful alcohol use and alcohol dependence. (4) Results were reported as 3D coordinates in a standard reference space.

For exclusion, the following criteria were defined: (1) review-studies, meta-analyses, and re-analyses, (2) region of interest analyses, small volume corrected results, and investigations with only partial brain coverage, (3) methodological studies and study protocols, (4) studies with small sample sizes (<10 per group) and (5) studies with statistical approaches not correcting for multiple comparisons or setting a minimum cluster extension as a statistical threshold for significance. Unlike conventional meta-analytical methods, (6) studies reporting null-findings could not be taken into account because they do not provide spatial coordinates, which are a prerequisite for coordinate-based meta-analyses. (7) Studies investigating patient groups with Korsakoff syndrome, with other primary psychopathology or comorbid other substance use disorder (except for tobacco).

Study selection and data extraction were performed independently by two investigators (CS and LM). Disagreements were solved by consensus with help of the supervising researcher (MM). The data extraction from the eligible studies included demographic sample characteristics and methodological characteristics as well as the resulting peak voxel coordinates. In addition, all studies were carefully checked for possible sample overlap. This step was also double-checked by CS and LM. Where data was missing or inconclusive in the original publications, we reached out to the corresponding authors to inquire about the supplementary information needed. In a few cases, we kindly received fast and helpful feedback (see Acknowledgements). Unfortunately, most of the contacted authors did not respond to our requests, and therefore, studies with missing necessary supplemental information were excluded.

Quality assessment was carried out in accordance with recommendations for analysis and reporting in neuroimaging [35] and guidelines for neuroimaging meta-analyses [27, 28]. Each included study was checked for a number of quality criteria such as sample and control group characteristics, information on MRI acquisition, and statistical analysis.

To estimate the robustness of the results to potential null findings, we used a calculation of the Fail-Safe N (FSN) adapted for ALE as described by Acar and colleagues [36]. Here, the FSN is defined as the amount of counterevidence (randomly generated study coordinates) that can be added to a meta-analysis before the results of that meta-analysis are altered.

### Anatomical likelihood estimation

The meta-analytical approach of anatomical likelihood estimation follows the principles of activation likelihood estimation (ALE) [37, 38], whereby the above-chance convergence among the reported coordinates of the individual studies gets detected. To ensure adequate power, the inclusion of 17–20 studies is recommended [28, 39].

For the preparation of the synthesis, the reported peak voxel coordinates and the sample sizes of the individual studies were manually extracted into a text file and served as input data for performing an ALE meta-analysis. In one case, where the peak voxel coordinates were not published and we requested them via personal correspondence, we received a NIfTI output file created by FMRIB Software Library (FSL) [40] Randomise tool (https://fsl.fmrib.ox.ac.uk/fsl/fslwiki/Randomise). For extraction of the most meaningful peak voxels, we used FSL v6.0 (https://fsl.fmrib.ox.ac.uk/fsl/fslwiki/FSL) on Windows 10 and the Cluster tool (https://fsl.fmrib.ox.ac.uk/fsl/fslwiki/Cluster), setting the threshold for the cluster level to *p* < 0.01. In three other studies, where the coordinates were specified in Talairach space, we used the Lancaster icbm2tal transform implemented in GingerALE v3.0.2 to transform to MNI space (http://www.brainmap.org/ale/) [41, 42]. The same version of GingerALE was used to perform the meta-analysis itself [37, 38, 43]. Prior to the actual meta-analytical calculations, four mask outliers were identified in the input data and were subjected to a plausibility check. In one case, for a coordinate reported from Yeh et al. [44] (x = 28, y = 52, z = 51), we suspected a missing negative sign. According to the anatomical label in the original publication, the sign of the y-coordinate was set to (−) as recommended in the User Manual for GingerALE 2.3 (https://brainmap.org/ale). Now the coordinate reads x = 28, y = −52, z = 51. Another outlier was located a few millimeters next to the brainstem, which could be caused by the transformation processes (Crespi et al. [45], x = −6, y = −9, z = −24). This coordinate, as well as the other two mask outliers (Harris et al. [46], x = 15, y = 46, z = −55 and x = −44, y = 18, z = −53), remained in the data set, because we could not clarify the exact position. The overall number of mask outliers was below the critical limit of 3% (see User Manual for GingerALE 2.3, https://brainmap.org/ale/). No sample overlap was identified within the studies eligible for inclusion and therefore data from each study was managed independently in the analysis. We combined the results from all contrasts (AUD < HC and AUD > HC), since ALE is testing independently from the direction of the effects [28]. If a sufficiently large number of individual studies was identified that used either DTI or VBM methods, appropriate subgroup analyses were performed in the course of the analysis.

**Table 1:** Demographic and clinical sample characteristics of the studies included in the ALE meta-analysis.

#	Source	AUD patients					Healthy controls	
		*n* (Fem.)	Age, *M* (*SD*)	Diagnosis (Diagnosis Criteria)	Duration of AUD in years *M* (*SD*)	Duration of Abstinence d/w/mo, *M* (*SD*)	*n* (Fem.)	Age, *M* (*SD*)
1	Asensio et al. [20]	24 (0)	35.62 (4.81)	Alcohol abuse (DSM-IV)	4.71 (2.93)	40.88 d (29.07)	24 (0)	31.91 (9.34)
2	Chanraud et al. [50]	26 (0)	47.7 (7.1)	Alcohol dependence (DSM-IV)	8 (6.3)	26.4 w (29.0)	24 (0)	45 (6.72)
3	Chumin et al. [51]	38 (7)	38.6 (8.1)	Alcohol dependence (DSM-IV)	n. a.	n. a.	19 (3)	37.8 (8.6)
4	Crespi et al. [45]	22 (9)	45.56 (7.99)	Alcohol dependence (DSM-IV)	10.11 (6.56)*	>10 d	18 (7)	45.11 (8.69)
5	Demirakca et al. [14]	50 (23)	46.6 (8.2)	Alcohol dependence (DSM-IV)	12.4 (7.4)	16.5 d (7.3)	66 (32)	45.0 (10.1)
6	De Santis et al. [52]	48 (0)	47.5 (1.4)	Alcohol use disorder (DSM-5)	n. a.	>3 d	36 (0)	41.7 (1.6)
7	Harris et al. [46]	15 (0)	48.3 (13.1)	Alcohol abuse or dependence (DSM-IV)	16.0 (8.0)	5.7 y (10.0)	15 (0)	56.4 (9.0)
8	Jang et al. [53]	20 (0)	43.5 (6)	Alcohol dependence (DSM-IV)	n. a.	7.8 d (6.5)	20 (0)	44.5 (7.4)
9	Konrad et al. [54]	24 (0)	48.5 (8.6)	Alcohol dependence (DSM-IV)	14.1 (10.2)	n. a.	23 (0)	47.4 (7.2)
10	Mechtcheriakov et al. [55]	22 (8)	53.6 (n. a.)	Alcohol addiction (ICD-10)	>10*	>10 d	22 (8)	53.7 (n. a.)
11	Monnig et al. [13]	10 (4)^C^	35.7 (7.8)^C^	Alcohol abuse or dependence (DSM-IV)	n. a.	>2 d^C^	15 (7)	32.9 (7.6)
		9 (2)R	36.4 (5.7)^R^			> 12 mR		
12	Pandey et al. [56]	30 (0)	41.42 (7.31)	Alcohol use disorder (DSM-IV)	n. a.	672.93 d (844.94)	30 (0)	27.44 (4.74)
13	Pitel et al. [57]	34 (6)	43.47 (8.36)	Alcohol dependence (DSM-IV)	16.09 (10.29)^+K^	12.67 d (6.94)^+K^	25 (14)	43.88 (11.24)
14	Sawyer et al. [58]	23 (0)	54.03 (11.39)	Alcohol abuse or dependence (DSM-IV)	>5	4.99 y (7.64)	19 (0)	49.85 (13.36)
15	Segobin et al. [19]	19 (2)	44.40 (6.07)	Alcohol dependence (DSM-IV)	15.15 (10.49)^m^	11.05 d (5.20)	20 (n. a.)	46.70 (4.25)
					8.22 (8.79)d			
16	Segobin et al. [59]	20 (4)	45.2 (8.1)	Alcohol dependence (DSM-IV)	18.3 (8.7)^m^	2.4 d (3.1)	14 (5)	45.4 (6.9)
					9.5 (6.7)d			
17	Yeh et al. [44]	11 (0)	47.0 (7.6)	Alcohol dependence (DSM-IV)	n. a.	6 d (3)	10 (0)	42.7 (9.4)
18	Zorlu et al. [60]	17 (0)	47.0 (7.0)	Alcohol dependence (DSM-IV)	12.2 (7.3)	17.1 d (1.8)	16 (0)	46.7 (7.5)

*AUD* alcohol use disorder, *Fem*. females, *d/w/mo* days/weeks/months, *n.a*. information not available.

^C+R^Here, the authors subdivided the AUD patients in “current” and “early remission” groups but also reported results of a combined contrast which we included in our analysis (s. suppl. Table S1).

^+K^These data refer to a general AUD group including patients with Korsakoff syndrome. In our analysis, we only included the data of the contrast results of the subgroup with uncomplicated alcoholism (s. suppl. Table S1).

^m^misuse and ^d^dependence.

*Duration of general alcohol consumption.

After preparing the input file, we set GingerALE’s thresholding options to cluster-level family-wise error correction (cFWE) with *p* < 0.001 as the cluster-forming threshold and *p* < 0.05 as the FWE threshold. This procedure ensures low susceptibility to false-positive results [28, 39]. We furthermore set preferences for the inclusion of WM in labeling the cluster analysis results.

The ALE procedure follows three steps, which we will only briefly describe here (see refs. [37, 38, 43] for detailed information). First, GingerALE tests for the spatial uncertainty of the reported coordinates by modeling them with a Gaussian function, thereby accounting for the sample size of each study. This is followed by the construction of a whole-brain map for each study, whereby each voxel gets a value assigned that is equal to the probability of WM volume or integrity alterations within it. Subsequently, these maps are merged across all studies resulting in an ALE image with ALE values representing the likelihood of these alterations and tested for statistical significance with correction for multiple comparisons.

Finally, the measure of effect represents an ALE image with ALE values that in turn indicates the likelihood that structural differences were found at least for one study at a given voxel. Outcome variables are MNI peak voxel coordinates of the resulting clusters of convergence as well as information on cluster sizes (mm³), cluster labels, and name and number of contributing studies.

The resulting ALE maps were visualized using Mango (v4.1, http://ric.uthscsa.edu/mango/mango.html) and MRIcroGL (v1.2.20210317, https://www.mccauslandcenter.sc.edu/mricrogl/).

### Assignment to human brain white matter atlas

Since the Talairach and Tournoux atlas [47], which is implemented in GingerALE, does not provide differentiated WM references [48], we decided to use an additional tractography-based atlas of human brain connections [49], implemented in MRIcroGL (v1.2.20210317, https://www.mccauslandcenter.sc.edu/mricrogl/), for labeling.

## Results

### Eligibility of studies

**Fig. 1:**
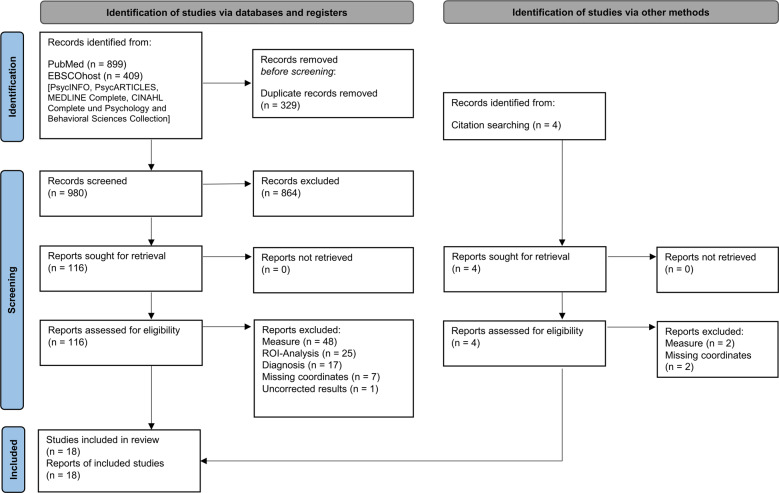
Stages of systematic literature search and selection: Flow diagram according to the PRISMA Guideline from Page et al. (2021) [34].

A systematic literature search revealed 18 studies eligible for inclusion [13, 14, 19, 20, 44–46, 50–60], entailing a total of 462 AUD patients and 416 healthy controls. The flow of information through the different phases of the review is depicted in Fig. 1. The main reason for exclusion refers to investigations of WM in AUD with other measures than DTI or VBM, for example, connectivity analysis (e.g., [61]) or WM signal hyperintensity analysis (e.g., [62]). Other common reasons for exclusion were region of interest analyses (e.g., [63–65]) and studies investigating subclinical samples not meeting AUD diagnosis criteria (e.g., [66]). Information about demographic and clinical sample characteristics from the included studies are presented in Table 1. Methodological features regarding data acquisition and analysis, as well as the source of reported peak-coordinates for each individual study can be found in supplementary Table S1.

The quality assessment for the included studies revealed high concordance with the selected criterions (Supplementary Table S2). General information regarding sample characteristics was given, but in a few cases specification of comorbidity and AUD duration or duration of abstinence appeared to be missing. Control groups were mainly matched and if not, in most cases, the authors integrated differences in age or sex as covariates in their analyses. Overall, MRI procedures and statistical analyses were described comprehensibly and missing information was accessible after personal correspondence.

### ALE results

**Fig. 2:**
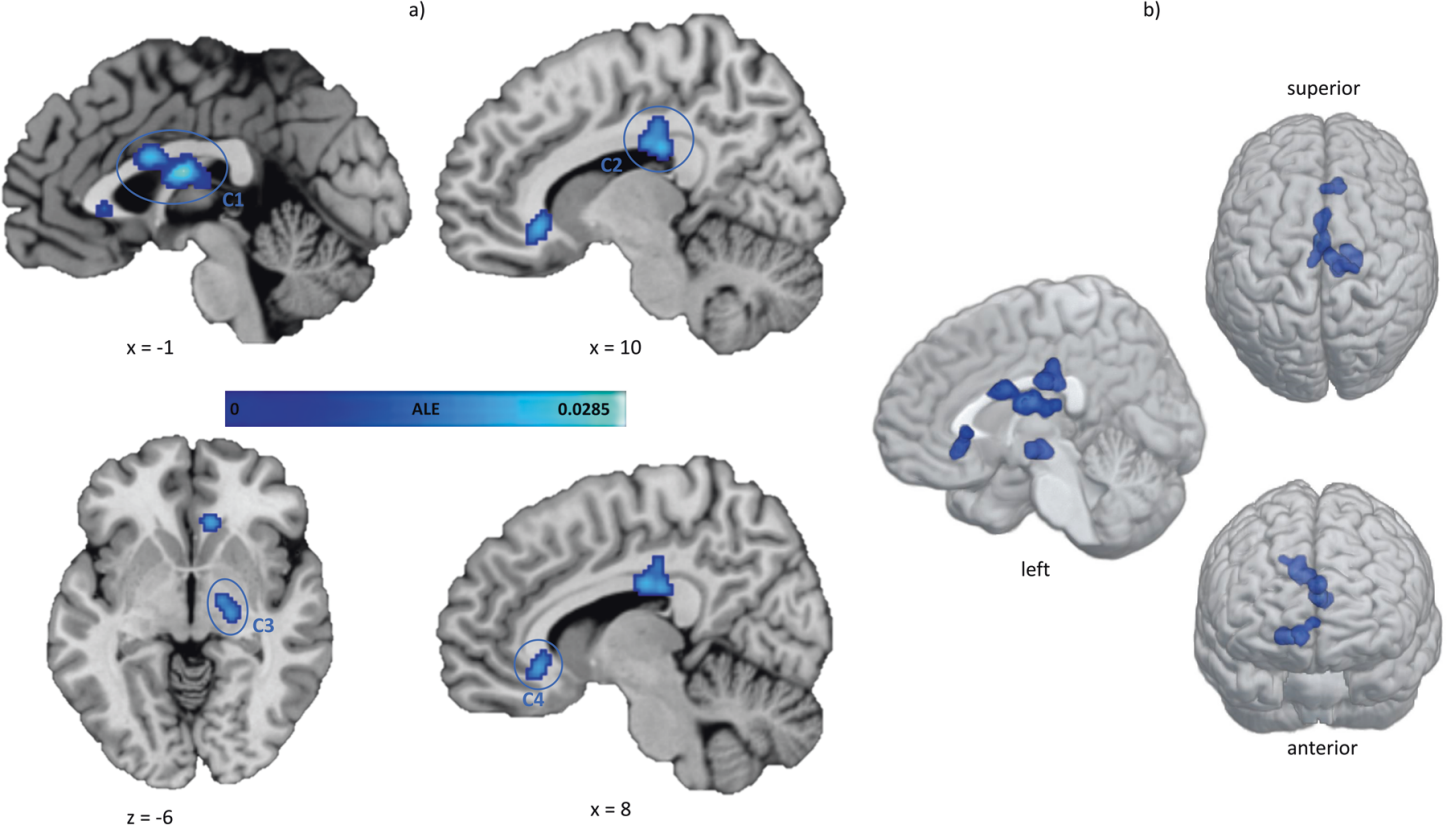
Results of the ALE meta-analysis. The highlighted clusters (C1–C4) represent significant convergence of white matter alterations in AUD patients compared to healthy controls. **a** Clusters are depicted on brain slices of an MNI standard brain. The color indicates the ALE value. **b** Spatial location and expansion of the ALE clusters depicted on a white matter glass brain. Cluster-forming threshold *p* < 0.001, FWE cluster level corrected at *p* < 0.05. x, y, and z values refer to coordinates in MNI space, for detailed MNI peak voxel coordinates of the ALE clusters see Table 2. This image was created with Mango (v4.1., http://ric.uthscsa.edu/mango/) and MRIcroGL (v1.2.20210317, https://www.mccauslandcenter.sc.edu/mricrogl/).

ALE revealed four significant clusters of convergent macro- and microstructural WM alterations in AUD patients compared to healthy controls, which are shown in Fig. 2. The largest cluster (C1) comprises parts of the midbody of the corpus callosum and the fornix in both hemispheres. The other clusters are mainly located in the right hemisphere and C2 also covers the posterior body of the corpus callosum with extension to the posterior cingulum bundle. The third cluster (C3) can be assigned to the right posterior limb of the internal capsule and the smallest and last cluster (C4) shows convergence in the genu of the corpus callosum with extension to the anterior cingulum bundle. The cluster sizes, peak-coordinates, and associated ALE values as well as the centers of mass are reported in Table 2.

**Table 2:** ALE clusters significant after cluster-level FWE correction for multiple comparisons.

		Peak voxel coordinates (MNI)							
Cluster #	Anatomical label^a^	x	y	z	ALE (*10^−2^)^b^	Cluster size (mm³)	Center of mass (x, y, z)	Contributing studies (%)^c^	Fail-Safe N(%)^c^
1	I Fornix	0	−8	16	2.84	2 200	−1, −4.4, 16.9	8 (44.4)	12 (66.6)
	I Corpus Callosum	0	6	22	2.24				
	L Fornix	−4	−16	12	1.78				
	L Fornix	−6	−20	14	1.75				
2	R Corpus Callosum	6	−18	28	2.23	1 776	10.1, −21, 29.4	7 (38.8)	51 (283.3)
	R Cingulum	10	−24	26	2.19				
	R Corpus Callosum	20	−24	36	1.53				
3	R Internal Capsule	16	−12	−8	2.10	1 064	18.8, −16.2, −8.6	5 (27.7)	6 (33.3)
	R Internal Capsule	20	−18	−8	2.01				
4	R Cingulum	10	28	−8	2.11	848	7.9, 27.4, −5.7	4 (22.2)	1 (5.5)
	R Corpus Callosum	4	26	0	1.77				

*I* interhemispheric, *L* left hemisphere, *R* right hemisphere, *x, y, z* coordinates provided in MNI space.

^a^Anatomical labeling according to the tractography-based atlas of human brain connections (Catani et al., 2008), as implemented in MRIcroGL (v1.2.20210317, https://www.mccauslandcenter.sc.edu/mricrogl).

^b^Maximum ALE value observed in the cluster.

^c^Ratio to the number of included experiments.

### Diagnostics of ALE results and post hoc analyses

Overall, 14 out of 18 included studies contributed to the identified clusters of convergence. Most of them contributed to C1 (eight studies), followed by C2 (seven studies), C3 (five studies), and C4 (four studies). The contributing studies as well as the number of contributing foci and their respective WM measures and contrasts are summarized in supplementary Table S3. The convergence of C1–C4 mainly emerges from foci referring to contrasts of reduced WM volume/density or structural integrity (e.g., lower fractional anisotropy (FA) and higher mean-, radial-, or axial diffusivity measures (MD, RD, AD) of WM fiber tracts in AUD patients. Only for C1, one foci refers to an opposite contrast of higher FA values and for C3, one foci refers to a contrast of higher WM volume. One of the contributing studies used a joint independent component analysis (jICA) procedure and did not report the direction of individual effects of the integrated coefficients (FA, MD, RD) [45]. The findings of this study can thus only be interpreted as a change in fiber integrity in AUD. Regarding the robustness of the meta-analytic results, the ALE clusters remained significant after adding 5 up to 283% noise studies (FSN). The calculated FSN values for each cluster are presented in the last column of Table 2.

### Sensitivity analysis

Because one of the integrated studies included patients whose duration of abstinence was significantly longer than in the other studies (Pandey et al., 2018) [56], we performed an additional analysis without this study to examine whether it biased the results. The results of this additional analysis were largely comparable to those of the main analysis (Supplementary Table S4 and Fig. S1).

### Exploratory subgroup analysis of DTI studies only

An exploratory subgroup analysis based on data from studies applying DTI methods only (*n* = 11), revealed four clusters of convergent microstructural WM alterations (Supplementary Table S5 and Fig. S2). Three clusters cover similar locations as the main analysis and comprise the left fornix, the right body of the corpus callosum with extension to the posterior cingulum bundle, and the right genu of the corpus callosum with extension to the anterior cingulum bundle. In contrast to the main analysis, the peak-coordinates of the studies in this subgroup did not show convergence in the right posterior limb of the internal capsule nor the midbody of the corpus callosum (C3 and parts of C1, see Fig. 1 and S2 for comparison). These clusters were characterized by a substantial proportion of VBM studies (3 of 5 and 3 of 8, respectively) and thus, the remaining DTI studies do not have sufficient weight to reach the significance threshold.

An additional cluster appears in the left hemisphere located in posterior parts of the left corpus callosum (C4 in Fig. S2) resulting from four contributing foci of four DTI studies [13, 45, 58, 59]. Both analyses were corrected at the cluster level using the FWE correction. This correction resulted in different minimum cluster sizes for the main and the sub-analysis and the diagnostics revealed that the corrected minimum cluster size for the main analysis with 18 studies is at least 824 mm^3^, whereas the corrected minimum cluster size in the sub-analysis with 11 studies is 688 mm^3^. The additional cluster of the sub-analysis with a cluster size of 744 mm^3^ thus only reaches the significance level in the “more liberal” sub-analysis but not in the main analysis.

## Discussion

Following a systematic literature search to identify VBM and DTI studies on the localization of WM alterations in AUD, we conducted a coordinate-based neuroimaging meta-analysis, which revealed four significant clusters of convergent macro- and microstructural WM changes. The clusters covered parts of the genu and the body of the corpus callosum with extensions to the fornix as well as the anterior and posterior cingulum bundle. Another cluster comprises the right posterior limb of the internal capsule. A similar pattern was observed when conducting the meta-analysis based on DTI studies only. The latter analysis revealed an additional cluster within the posterior parts of the left corpus callosum.

In this meta-analysis, we included contrasts that map both, reductions and increases in WM volume/density or WM integrity. In addition, we were able to integrate results from studies that used non-directional methods such as joint ICA [45]. This is possible because the ALE method itself tests directionally independent since it is not based on effect sizes [28], which is particularly useful in the context of clinical samples, where the effects may vary over the course of the disorder (e.g. as a function of the duration of abstinence or changes in the proportion of gray to white matter) [23, 26]. A close inspection of the contributing foci revealed that the ALE convergence clusters identified in our meta-analysis are largely based on studies that report a reduction of WM volume, density, or integrity in AUD patients.

The identified regions cover the anterior part of the body of the corpus callosum, which connects premotor and motor regions of both hemispheres and the genu of the corpus callosum connects large portions of the prefrontal cortices [67, 68]. Another cluster of WM structure changes was located in the cingulum bundle which interconnects frontal, parietal, and medial temporal brain regions as well as subcortical nuclei to the cingulate gyrus [69]. The posterior limb of the internal capsule comprises fibers that connect visual, auditory, somatosensory, and motor regions [70, 71]. Regarding the functional consequences, even subtle alterations in areas of the corpus callosum and cingulate bundle can lead to numerous functional impairments, as they represent important WM structures with interhemispheric and intrahemispheric pathways [69, 72], respectively. WM alterations within these structures could explain to some extent the decline in motor, affective, perceptive, and cognitive functions related to AUD [73]. For example, AUD-related alterations in callosal fibers are associated with changes in executive function [45] and decision-making deficits [60]. In particular, reduced integrity of the genu of the corpus callosum could be associated with poorer working memory performance [74]. However, another study could not find a direct correlation between the integrity of the callosal fibers and cognitive performance in AUD, but a correlation between the integrity of the cingulum, executive functions, and psychomotor performance [54]. Furthermore, the cingulum bundle, as well as the fornix form two major fiber tracts of the limbic system, and their degradation, might promote deficits in emotion regulation processes [75]. For example, lower FA in frontoparietal and corticolimbic networks as well as in deep WM structures, like the internal capsule, has been linked to higher alcohol cue reactivity in heavy drinkers [76]. In addition, lower visuospatial memory performance, another neurobehavioral consequences of heavy chronic alcohol consumption (e.g., [77] for review), has been associated with reduced commissural FA in AUD patients compared to controls [44]. Furthermore, the corpus callosum and the cingulum have been found to interconnect key nodes of large-scale brain networks, such as the default mode network, and the body of the corpus callosum connects left and right hemispheric parts of the primary sensorimotor network [78].

In summary, the above examples point towards an association of WM changes in AUD patients identified by this meta-analysis and several behavioral impairments. Together with previously reported GM reductions in AUD [18], they may explain the deterioration of a wide range of motor, cognitive, affective, and perceptual functions in individuals with AUD. However, the behavioral interpretation of our results is still speculative as it is based on a few single studies and as we could not apply a data-driven approach that is comparable to the workflows via the BrainMap database for GM data [79]. Thus, further studies are needed to explore the behavioral meaning of the WM changes in the identified clusters. Despite the clear meta-analytical evidence for substantial changes of WM in AUD, the underlying molecular or cellular mechanisms remain unexplored. With respect to the DTI measures, it is possible that alcohol-induced reduction of myelin may be caused by inflammatory or epigenetic processes [8, 80]. In addition, a reduction of axonal fibers may have occurred due to direct alcohol toxicity to neuronal and glial cells. Lastly, changes in the chemical composition of fibers, as well as the ratio between axonal fibers, oligodendrocytes, and other glial cells in AUD patients, may account for observed effects (e.g., [12, 80] for review). Together, chronic high alcohol consumption has been shown to cause multiple molecular and systemic changes that may account for observed WM alterations. In this regard, it is interesting to note structural damages observed in AUD patients partially regenerated during prolonged abstinence [63]. Alternatively or in addition, a preexisting state of WM alterations may also be the cause or at least facilitate the development of AUD, for example in individuals with a family history of AUD [81]. Furthermore, it should be considered that the largest cluster of the main analysis and the second-largest cluster of the DTI sub-analysis cover the fornix. The fornix is a thin structure located close to the ventricles, which makes it particularly susceptible to partial volume effects [82]. This effect occurs when in a voxel the signal is not only represented by one substance (e.g., WM) but is also confounded by other substances such as cerebrospinal fluid (CSF). The mixing of signals leads to an increase in diffusivity measures and a decrease in anisotropy measures and is particularly pronounced when small, thin structures are affected and when groups with brain atrophy are compared with healthy controls [82]. Both are evident in the integrated studies. For adequate correction of the partial volume effect, voxel-wise CSF contamination correction, such as free water elimination, is recommended [82, 83]. None of the integrated studies explicitly mentioned that such a correction was performed. Despite the clear meta-analytic evidence, the ALE cluster covering the fornix should therefore be interpreted with caution, as there is a possibility that the results of the contributing studies may be biased by the partial volume effect.

Finally, it should be noted that the results of our analysis are not consistent with those of another recent meta-analysis [26], where a pattern of convergent WM volumetric alterations in AUD in the corticospinal tract and anterior thalamic radiation has been identified. However, this meta-analysis was not explicitly designed to investigate WM alterations in AUD but reported respective findings as a part of a sub-analysis in a broader context based on four studies only.

Although our work fills a gap regarding lacking meta-analytical evidence on WM alterations in AUD by strictly following state-of-the-art guidelines for neuroimaging meta-analyses (for checklist see Supplementary Table S6), it is subject to several limitations. First, even with pre-specified inclusion and exclusion criteria, the integrated studies are heterogeneous in certain aspects (e.g., sample characteristics like sex distribution, see Table 1). Second, in two studies the AUD samples comprised participants who reported co-consumption of other substances (e.g., [13, 56]). Furthermore, there is a high range of overall abstinence durations between the studies included. Therefore, meta-regression could be informative but unfortunately are not feasible in ALE analyses. Third, the ALE method is insensitive to non-significant results [28] and is thus susceptible to potentially unobserved publication bias. To account for these uncertainties, we calculated a Fail-Safe N, modified for ALE analyses [36], for each of the ALE clusters. Respective results indicate that most clusters show stable effects against additional noise studies and are not driven by a few very dominant studies.

In conclusion, we identified four clusters of convergent macro- and microstructural WM alterations in patients with AUD through ALE meta-analysis. The resulting clusters have been assigned to key brain structures of the cingulum, corpus callosum, fornix, and internal capsule. Respective reductions in WM volume and axonal integrity may reflect either permanent or partly transient changes in AUD patients that have been associated with several neuropsychological deficits (e.g., decision making and emotion regulation) in functional neuroimaging studies [60, 76]. Future research is needed to provide a more accurate behavioral assessment of the identified WM clusters and to examine the extent of reversibility of alcohol-related WM changes.

## Supplementary information

Supplementary Material

PRISMA_2020_checklist
