# Supplementary material for: A coordinate-based meta-analysis of white matter alterations in patients with alcohol use disorder
Source: Transl Psychiatry. 2022 Jan 27;12:40. doi: 10.1038/s41398-022-01809-0 (PMC8795454; doi:10.1038/s41398-022-01809-0)
Supplement: Supplementary file 1 — Supplementary Material [file 41398_2022_1809_MOESM1_ESM.pdf]

Supplementary Material

A coordinate-based meta-analysis of white matter alterations in patients with alcohol use disorder

Carolin Spindler (M.Sc.)[1,2], Louisa Mallien (M.Sc.)[3], Sebastian Trautmann (Ph.D.)[1,4], Nina Alexander (Ph.D.)[1,5,6], Markus Muehlhan (Ph.D.)[1,2*].

[1] Department of Psychology, Faculty of Human Sciences, Medical School Hamburg, Am Kaiserkai 1, 20457 Hamburg, Germany

[2] ICAN Institute for Cognitive and Affective Neuroscience, Medical School Hamburg, Am Kaiserkai 1, 20457 Hamburg, Germany

[3] Department of Human Medicine, Faculty of Medicine, Medical School Hamburg, Am Kaiserkai 1, 20457 Hamburg, Germany

[4] ICPP Institute for Clinical Psychology and Psychotherapy, Medical School Hamburg, Am Kaiserkai 1, 20457 Hamburg, Germany

[5] Department of Psychiatry and Psychotherapy, Philipps University Marburg, Rudolf-Bultmann-Str. 8, 35039 Marburg, Germany

[6] Center for Mind, Brain and Behavior, Philipps University Marburg, Hans-Meerwein-Str. 6, 35032 Marburg, Germany

*Corresponding author:
Markus Muehlhan, Prof. Dr. rer. nat. (Ph.D.)
Department of Psychology
Faculty of Human Science
Medical School Hamburg MSH
Am Kaiserkai 1
D-20457 Hamburg, Germany
Phone: +49 40 36 12 26 49324
Fax: +49 40 36 12 26 430
E-Mail: markus.muehlhan@medicalschool-hamburg.de

Table S1
Methodological characteristics of the studies included in the ALE meta-analysis.

#	Source	Field strength	Method	Smooth Kernel	Diffusion Gradient Directions	Software	Correction	Threshold	Covariates	Measure	Contrasts	No. of Foci	Ref. Space	Source of coordinates
1	Asensio et al. (2016)	1.5 T	VBM	8mm	-	SPM5	k ≥ 50	p < 0.05	age, total GMV and WMV	Volume	AUD > HC<br>AUD < HC	2<br>2	MNI	Table 2
2	Chanraud et al. (2007)	1.5 T	VBM	8mm	-	SPM2	FDR<br>k ≥ 50	p < 0.005	age, education, smoking	Volume	AUD < HC	12	MNI	Table 3
3	Chumin et al. (2018)	3 T	DWI/TBSS	-	48	FSL	TFCE, FWE	p < 0.05	-	FA	AUD < HC	33	MNI	Table S1
4	Crespi et al. (2019)	3 T	DTI/TBSS (jICA)	-	81	FSL	k ≥ 10	p = 0.004	-	FA, AD, MD, RD	n. a.	113	MNI	Table S2-4
5	Demirakca et al. (2011)	1.5 T	VBM	8mm	-	SPM8	FWE	p < 0.05	age, sex, TIV	Volume	AUD < HC	7	MNI	Table S5
6	De Santis et al. (2019)	3 T	DTI/TBSS	-	41	Explore DTI, FSL	TFCE, FWE	p < 0.05	age	FA,<br>MD	AUD < HC<br>AUD > HC	7<br>8	MNI	p . c.
7	Harris et al. (2008)	3 T	DTI	8mm	6	SPM, FSL	k ≥ 5$^{p. c.}$	p < 0.01	age	FA	AUD < HC<br>AUD > HC	5<br>1	TAL	Table 3
8	Jang et al. (2007)	3 T	VBM	8mm	-	ANA-LYZE SPM2	FDR<br>k ≥ 100	p < 0.05	-	Density	AUD < HC	6	TAL	Table 3
9	Konrad et al. (2012)	1.5 T	DTI/TBSS	6mm	6	MRIcro<br>FSL<br>SPM5	FWE	p < 0.05	-	FA	AUD < HC	8	MNI	Table 2
10	Mechtcheriakov et al. (2007)	1.5 T	VBM	10mm	-	SPM2	FDR	p < 0.05	global mean voxel values, TIV	Volume	AUD < HC	3	MNI	Table 2
11	Monnig et al. (2013)	3 T	DTI/TBSS	-	30	FSL	TFCE<br>k ≥ 100	p < 0.05	-	FA	AUD^{C+R} < HC	3	MNI	Table 2

Table S1 continued

12	Pandey et al. (2018)	3 T	DTI	-	30	FreeSurfer	FWE	$p < 0.05$	age	FA, MD, AD, RD	AUD < HC<br>AUD > HC	10	MNI	Table 2
13	Pitel et al. (2012)	1.5 T	VBM	10mm	-	SPM5	FDR<br>k ≥ 200	$p < 0.01$	age, sex	Volume	AUD < HC	23	MNI	p. c.
14	Sawyer et al. (2018)	3 T	DTI/TBSS	-	60	FSL	TFCE, FWE	$p < 0.05$	-	FA	AUD < HC	1	TAL	Table 2
15	Segobin et al. (2014)	1.5 T	VBM	8mm	-	SPM5	FDR<br>k ≥ 500	$p < 0.01$	age	Volume	AUD < HC	4	MNI	Figure 1
16	Segobin et al. (2015)	3 T	DTI/TBSS	-	32	FSL	TFCE, FWE	$p < 0.05$	age	FA	AUD < HC	48	MNI	p. c.
17	Yeh et al. (2009)	1.5 T	DTI/TBSS	n. a.	6	FSL	FDR<br>k ≥ 200	$p < 0.05$	age	FA, MD<br>AD, RD	AUD < HC<br>AUD > HC	62	MNI	Table 1
18	Zorlu et al. (2013)	1.5 T	DTI/TBSS	-	100	FSL	TFCE	$p < 0.05$	-	FA, AD,RD	AUD < HC<br>AUD > HC	4	MNI	Table 2

n.a.= information not available, VBM= voxel based morphometry, DTI= diffusion tensor imaging, TBSS= tract based spatial statistics, jICA= joint independent component analysis, SPM= Statistical Parametric Mapping, FSL= FMRIB Software Library, k= cluster size in voxels, FDR= False Discovery Rate, FWE= Family Wise Error Correction, TFCE= Threshold-free Cluster Enhancement , GMV= Gray matter volume, WMV= White matter volume, TIV= Total intracranial volume, FA= fractional anisotropy, AD= axial diffusivity, MD= mean diffusivity, RD= radial diffusivity, [C+R] the authors subdivided the AUD patients in “current” and “early remission” groups but also reported results of a combined contrast which we included in our analysis, MNI= Montreal Neurological Institute, TAL= Talairach, p.c.= personal correspondence.

Table S2: Quality assessment for studies included in the ALE meta-analysis

Quality criterion	Study number																	
	1	2	3	4	5	6	7	8	9	10	11	12	13	14	15	16	17	18
Report of clinical sample characteristics																		
• Sample size (≥10)	✓	✓	✓	✓	✓	✓	✓	✓	✓	✓	✓	✓	✓	✓	✓	✓	✓	✓
• Mean age and sex distribution	✓	✓	✓	✓	✓	✓	✓	✓	✓	✓	✓	✓	✓	✓	✓	✓	✓	✓
• Diagnosis criteria	✓	✓	✓	✓	✓	✓	✓	✓	✓	✓	✓	✓	✓	✓	✓	✓	✓	✓
• Comorbidity	✓	✓	✓	✓	✓	✓	✓	✓	✓	✓	✓	?	✓	?	✓	✓	✓	✓
• Duration of AUD	✓	✓	×	✓	✓	×	✓	×	✓	✓	×	×	✓	✓	✓	✓	×	✓
• Duration of abstinence	✓	✓	×	✓	✓	✓	✓	✓	×	✓	✓	✓	✓	✓	✓	✓	✓	✓
Description of control group																		
• Sample size (≥10)	✓	✓	✓	✓	✓	✓	✓	✓	✓	✓	✓	✓	✓	✓	✓	✓	=10	✓
• Matched on age and sex	✓	✓	✓	✓	✓	× ✓	✓	✓	✓	✓	✓ ×	× ✓	✓ ×	✓	✓ ?	✓ x	✓	✓
Information given on MRI procedures																		
• Imaging parameters	✓	✓	✓	✓	✓	✓	✓	✓	✓	✓	✓	✓	✓	✓	✓	✓	✓	✓
• Acquisition methods	✓	✓	✓	✓	✓	✓	✓	✓	✓	✓	✓	✓	✓	✓	✓	✓	✓	✓
• Whole brain coverage	✓	✓	✓	✓	✓	✓	✓	✓	✓	✓	✓	✓	✓	✓	✓	✓	✓	✓
• Standard reference space	✓	✓	✓	✓	✓	✓	✓	✓	✓	✓	✓	✓	✓	✓	✓	✓	✓	✓
• Image processing	✓	✓	✓	✓	✓	✓	✓	✓	✓	✓	✓	✓	✓	✓	✓	✓	✓	✓
Information given on statistical analysis																		
• Modelling approach	✓	✓	✓	✓	✓	✓	✓	✓	✓	✓	✓	✓	✓	✓	✓	✓	✓	✓
• Software used	✓	✓	✓	✓	✓	✓	✓	✓	✓	✓	✓	✓	✓	✓	✓	✓	✓	✓
• Correction for multiple comparisons	×*	✓	✓	✓	✓	✓	×*/**	✓	✓	✓	✓	✓	✓	✓	✓	✓	✓	✓
Presentation of results																		
• Table of peak coordinates	✓	✓	✓	✓	✓	×**	✓	✓	✓	✓	✓	✓	×**	✓	✓	×**	✓	✓

✓ = study meets criterion, ? = unclear/information is not given, × = study does not meet criterion, * = setting minimum cluster sizes instead, ** = information received through personal correspondence

Next page for study references 1/2

Study References

1 Asensio S, Morales JL, Senabre I, Romero MJ, Beltran MA, Flores-Bellver M *et al.* Magnetic resonance imaging structural alterations in brain of alcohol abusers and its association with impulsivity. *Addict Biol* 2016; **21**: 962–971.

2 Chanraud S, Martelli C, Delain F, Kostogianni N, Douaud G, Aubin H-J *et al.* Brain Morphometry and Cognitive Performance in Detoxified Alcohol-Dependents with Preserved Psychosocial Functioning. *Neuropsychopharmacology* 2007; **32**: 429–438.

3 Chumin EJ, Goñi J, Halcomb ME, Durazzo TC, Dzemidzic M, Yoder KK. Differences in White Matter Microstructure and Connectivity in Nontreatment-Seeking Individuals with Alcohol Use Disorder. *Alcohol Clin Exp Res* 2018; **42**: 889–896.

4 Crespi C, Galandra C, Manera M, Basso G, Poggi P, Canessa N. Executive Impairment in Alcohol Use Disorder Reflects Structural Changes in Large-Scale Brain Networks: A Joint Independent Component Analysis on Gray-Matter and White-Matter Features. *Front Psychol* 2019; **10**. doi:10.3389/fpsyg.2019.02479.

5 Demirakca T, Ende G, Kämmerer N, Welzel-Marquez H, Hermann D, Heinz A *et al.* Effects of Alcoholism and Continued Abstinence on Brain Volumes in Both Genders. *Alcohol Clin Exp Res* 2011; **35**: 1678–1685.

6 De Santis S, Bach P, Pérez-Cervera L, Cosa-Linan A, Weil G, Vollstädt-Klein S *et al.* Microstructural White Matter Alterations in Men With Alcohol Use Disorder and Rats With Excessive Alcohol Consumption During Early Abstinence. *JAMA Psychiatry* 2019; **76**: 749–758.

7 Harris GJ, Jaffin SK, Hodge SM, Kennedy D, Caviness VS, Marinkovic K *et al.* Frontal White Matter and Cingulum Diffusion Tensor Imaging Deficits in Alcoholism. *Alcohol Clin Exp Res* 2008; **32**: 1001–1013.

8 Jang D-P, Namkoong K, Kim J-J, Park S, Kim I-Y, Kim SI *et al.* The relationship between brain morphometry and neuropsychological performance in alcohol dependence. *Neurosci Lett* 2007; **428**: 21–26.

9 Konrad A, Vucurevic G, Lorscheider M, Bernow N, Thümmel M, Chai C *et al.* Broad Disruption of Brain White Matter Microstructure and Relationship with Neuropsychological Performance in Male Patients with Severe Alcohol Dependence. *Alcohol Alcohol* 2012; **47**: 118–126.

10 Mechtcheriakov S, Brenneis C, Egger K, Koppelstaetter F, Schocke M, Marksteiner J. A widespread distinct pattern of cerebral atrophy in patients with alcohol addiction revealed by voxel-based morphometry. *J Neurol Neurosurg Psychiatry* 2007; **78**: 610–614.

11 Monnig MA, Caprihan A, Yeo RA, Gasparovic C, Ruhl DA, Lysne P *et al.* Diffusion Tensor Imaging of White Matter Networks in Individuals with Current and Remitted Alcohol Use Disorders and Comorbid Conditions. *Psychol Addict Behav J Soc Psychol Addict Behav* 2013; **27**: 455–465.

12 Pandey AK, Ardekani BA, Kamarajan C, Zhang J, Chorlian DB, Byrne KN-H *et al.* Lower Prefrontal and Hippocampal Volume and Diffusion Tensor Imaging Differences Reflect Structural and Functional Abnormalities in Abstinent Individuals with Alcohol Use Disorder. *Alcohol Clin Exp Res* 2018; **42**: 1883–1896.

13 Pitel A-L, Chételat G, Berre APL, Desgranges B, Eustache F, Beaunieux H. Macrostructural abnormalities in Korsakoff syndrome compared with uncomplicated alcoholism. *Neurology* 2012; **78**: 1330–1333.

14 Sawyer KS, Maleki N, Papadimitriou G, Makris N, Oscar-Berman M, Harris GJ. Cerebral white matter sex dimorphism in alcoholism: a diffusion tensor imaging study. *Neuropsychopharmacology* 2018; **43**: 1876–1883.

15 Segobin SH, Chételat G, Le Berre A-P, Lannuzel C, Boudehent C, Vabret F *et al.* Relationship between brain volumetric changes and interim drinking at six months in alcohol-dependent patients. *Alcohol Clin Exp Res* 2014; **38**: 739–748.

16 Segobin S, Ritz L, Lannuzel C, Boudehent C, Vabret F, Eustache F *et al.* Integrity of white matter microstructure in alcoholics with and without Korsakoff's syndrome. *Hum Brain Mapp* 2015; **36**: 2795–2808.

17 Yeh P-H, Simpson K, Durazzo TC, Gazdzinski S, Meyerhoff DJ. Tract-Based Spatial Statistics (TBSS) of diffusion tensor imaging data in alcohol dependence: abnormalities of the motivational neurocircuitry. *Psychiatry Res* 2009; **173**: 22–30.

18 Zorlu N, Gelal F, Kuserli A, Cenik E, Durmaz E, Saricicek A *et al.* Abnormal white matter integrity and decision-making deficits in alcohol dependence. *Psychiatry Res* 2013; **214**: 382–388.

Table S3
Summarized information on measures and contrasts regarding the contributing studies to ALE clusters of convergence.

ALE Cluster	Contributing experiments (14/18)	No. of contributing Foci	WM Measure	Contrast
C1	Chanraud et al. (2007)	1	Volume	AUD < HC
	Crespi et al. (2019)	13	FA<br>RD<br>AD<br>MD	n. a.*
	De Santis et al. (2019)	1	MD	AUD > HC
	Jang et al. (2007)	1	Density	AUD < HC
	Monnig et al. (2013)	1	FA	AUD < HC
	Pandey et al. (2018)	1	FA	AUD > HC
	Pitel et al. (2012)	2	Volume	AUD < HC
	Segobin et al. (2015)	1	FA	AUD < HC
C2	Chumin et a. (2018)	2	FA	AUD < HC
	Crespi et al. (2019)	3	FA	n. a.*
	Demirakca et al. (2011)	1	Volume	AUD < HC
	De Santis et al. (2019)	1	FA	AUD < HC
	Konrad et al. (2012)	1	FA	AUD < HC
	Pandey et al. (2018)	1	FA<br>RD	AUD < HC<br>AUD > HC
	Yeh et al. (2009)	2	RD<br>MD	AUD > HC<br>AUD > HC
C3	Asensio et al. (2016)	1	Volume	AUD > HC
	Crespi et al. (2019)	3	FA<br>AD<br>MD	n. a.*
	Demirakca et al. (2011)	1	Volume	AUD < HC
	Pitel et al. (2012)	1	Volume	AUD < HC
	Segobin et al. (2015)	1	FA	AUD < HC
C4	Crespi et al. (2019)	6	RD<br>AD<br>MD	n. a.*
	Demirakca et al. (2011)	1	Volume	AUD < HC
	Segobin et al. (2015)	2	FA	AUD < HC
	Zorlu et al. (2013)	1	FA<br>RD	AUD < HC<br>AUD > HC
	**Not contributing (4/18)**			
	Harris et al. (2008)			
	Mechtcheriakov et al. (2007)			
	Sawyer et al. (2018)			
	Segobin et al. (2014)			

FA= fractional anisotropy, RD= radial diffusivity, AD= axial diffusivity, MD= mean diffusivity, n. a.*= information not available, combined contrasts within joined independent component analysis.

Table S4

ALE clusters significant after exclusion of data from a sample with longer abstinence duration (Pandey et al., 2018), cluster-level FWE corrected.

		Peak Voxel Coordinates (MNI)						
Cluster #	Anatomical Label[a]	x	y	z	ALE ($*10^{-2}$)[b]	Cluster Size (mm³)	Center of mass (x, y, z)	Contributing Studies (%)
1	L Fornix	0	-8	16	2.84	2,048	-0.6,-2.9,17.6	7 (41.2)
	I Corpus Callosum	0	6	22	2.24			
	L Fornix	-6	-20	14	1.62			
2	R Corpus Callosum	6	-18	28	2.23	1,640	9.3,-20.8,28.9	6 (35.3)
	R Cingulum	10	-24	26	2.19			
3	R Internal Capsule	16	-12	-8	2.10	1,088	18.7,-16.2,-8.6	5 (29.4)
	R Internal Capsule	20	-18	-8	2.01			
4	R Cingulum	10	28	-8	2.11	912	7.8,27.4,-5.6	4 (23.5)
	R Corpus Callosum	4	26	0	1.77			

R, right hemisphere; I, interhemispheric; L, left hemisphere; x, y, z coordinates provided in MNI space.
a Anatomical labelling according to the tractography based atlas of human brain connections (Catani et al., 2008), as implemented in MRIcroGL (v1.2.20210317, https://www.mccauslandcenter.sc.edu/mricrogl).
b Maximum ALE value observed in the cluster.

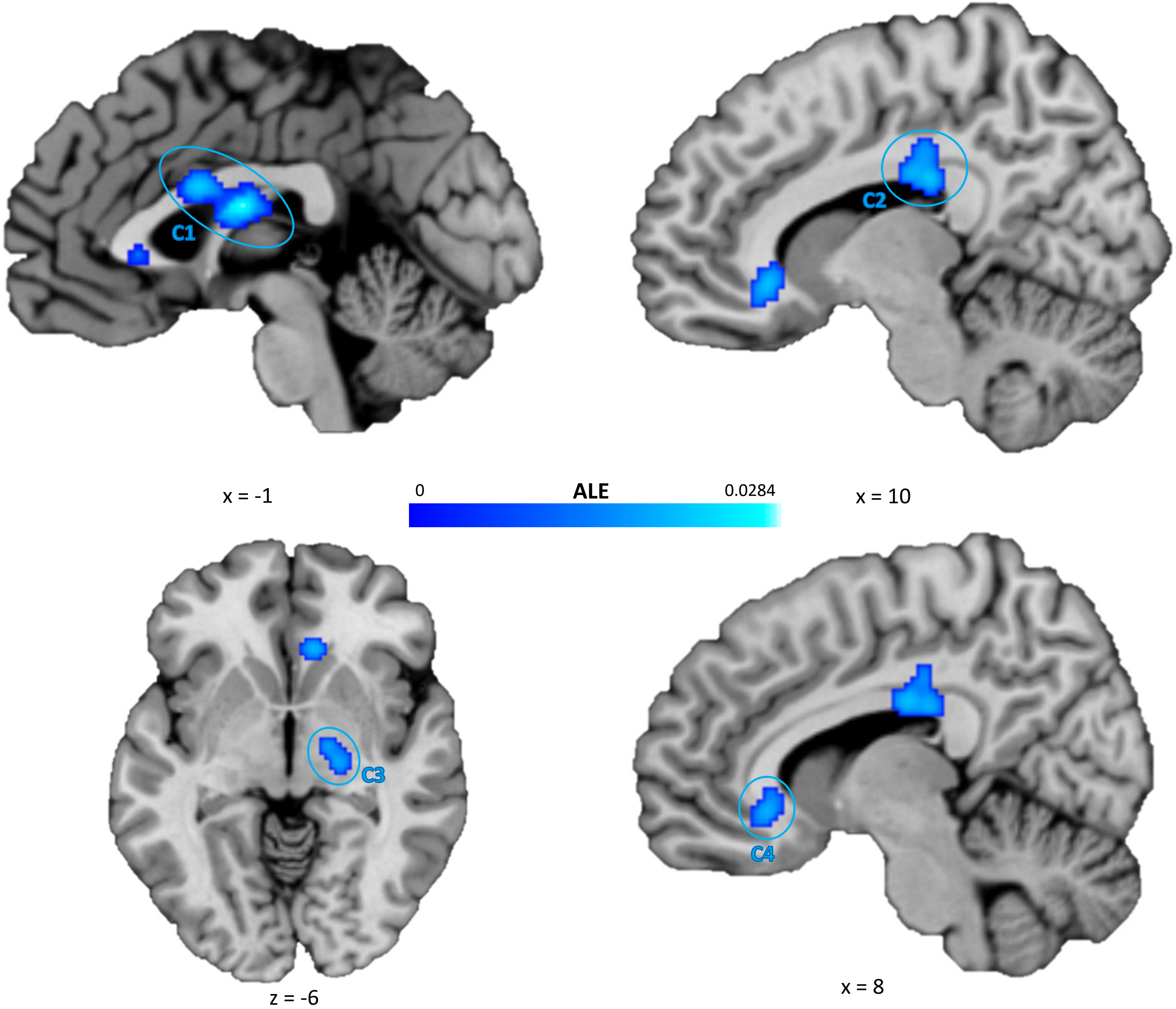

Fig. S1: ALE clusters significant after exclusion of data from a sample with longer abstinence duration (Pandey et al., 2018). The highlighted clusters (C1-C4) represent significant convergence about white matter alterations in AUD patients compared to healthy controls. Clusters are depicted on brain slices of an MNI standard brain. Color indicates ALE value. Cluster-forming threshold $p < 0.001$, family wise error (FWE) cluster level corrected at $p < 0.05$. x, y and z values refer to coordinates in MNI space, for detailed MNI peak voxel coordinates of the ALE clusters see table S4. This image was created with Mango (v4.1., http://ric.uthscsa.edu/mango/).

Table S5

Exploratory subgroup analysis of DTI studies investigating WM differences in AUD: ALE clusters significant after cluster-level FWE correction for multiple comparisons.

Cluster #	Anatomical Label[a]	Peak Voxel Coordinates (MNI) x	y	z	ALE ($*10^{-2}$)[b]	Cluster Size (mm^3)	Center of mass (x, y, z)	Contributing Studies (%)
1	R Corpus Callosum	6	-18	28	2.16	1 704	9.7, -21.2, 30.1	6 (54.5)
	R Cingulum	10	-26	26	1.80			
	R Cingulum	10	-24	36	1.60			
	R Corpus Callosum	20	-24	36	1.51			
2	I Fornix	0	-8	16	2.52	1 320	-2.3, -11.9, 14.3	5 (45.4)
	L Fornix	-4	-16	12	1.77			
	L Fornix	-6	-20	14	1.75			
3	R Corpus Callosum	10	28	-10	1.86	800	6.5, 27.6, -5.5	3 (27.3)
	R Corpus Callosum	4	26	0	1.75			
4	L Corpus Callosum	-28	-58	18	2.39	744	-28.2, -57, 17.6	4 (36.3)

R, right hemisphere; I, interhemispheric; L, left hemisphere; x, y, z coordinates provided in MNI space.
a Anatomical labelling according to the tractography based atlas of human brain connections (Catani et al., 2008), as implemented in MRIcroGL (v1.2.20210317, https://www.mccauslandcenter.sc.edu/mricrogl).
b Maximum ALE value observed in the cluster.

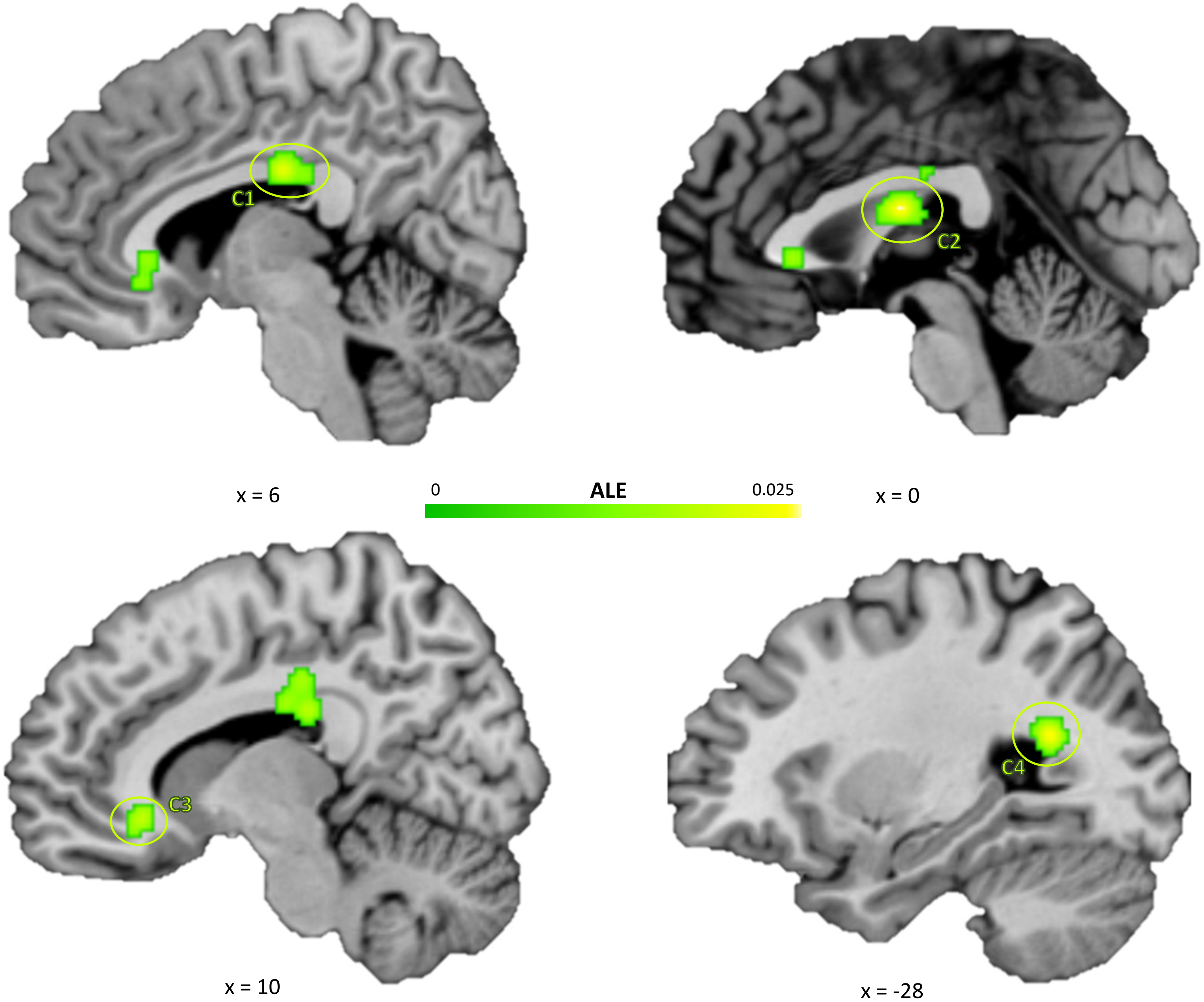

Fig. S2: Exploratory ALE subgroup analysis of DTI studies (n=11) investigating WM differences in AUD. The highlighted clusters (C1-C4) represent significant convergence about microstructural white matter alterations in AUD patients compared to healthy controls. Clusters are depicted on brain slices of an MNI standard brain. Color indicates ALE value. Cluster-forming threshold $p < 0.001$, family wise error (FWE) cluster level corrected at $p < 0.05$. x, y and z values refer to coordinates in MNI space, for detailed MNI peak voxel coordinates of the ALE clusters see table S5. This image was created with Mango (v4.1., http://ric.uthscsa.edu/mango/).

Table S6
Checklist for neuroimaging meta-analysis according to Müller et al. (2018).

The research question is specifically defined	YES, and it includes the following contrast:<br><br>whole brain macro- and microstructural WM alterations in AUD vs. whole brain macro- and microstructural WM alterations in HCs<br><br>→ ***Introduction, last paragraph***
The literature search was systematic	YES, it included the following keywords in the following databases:<br><br>**Keywords:**<br>(alcohol misuse OR alcoholism OR alcohol drinking OR drinking behavior OR binge drinking OR alcoholics OR alcohol use disorder OR alcohol dependence OR alcohol addiction OR chronic alcoholic intoxication OR alcohol abuse) AND (white matter OR white brain matter OR cerebellar white matter OR white matter integrity) AND (diffusion tensor* OR DTI OR magnetic resonance imaging OR tractography OR mean diffusivity OR axial diffusivity OR radial diffusivity OR fractional anisotropy OR structural connectivity OR structural changes OR structural MRI OR voxel-based morphometry OR VBM)<br><br>**Databases:** PubMed and EBSCO hosted PsycINFO, PsycARTICLES, MEDLINE Complete, CINAHL Complete and Psychology and Behavioral Sciences Collection databases (up to January 18, 2021)<br><br>→ ***Methods section: Literature Search, Study Selection and Data Extraction***
Detailed inclusion an exclusion criteria are included	YES, and reasons of non-standard criterion were:<br><br>Exclusion of studies reporting null-findings, they cannot be taken into account because they do not provide spatial coordinates, which are a prerequisite for the coordinate-based meta-analytical approach.<br><br>→ ***Methods section: Literature Search, Study Selection and Data Extraction***
Sample overlap was taken into account	NO sample overlap was identified within the studies eligible for inclusion and therefore data from each study was managed as independent experiment in the analysis.

Table S6 continued

	→ ***Methods section: Anatomical Likelihood Estimation***
All experiments use the same search coverage (state how brain coverage is assessed and how small volume corrections and conjunctions are taken into account)	YES, the search coverage is the following:<br><br>whole brain coverage only, verified via details of the scanner parameters provided in the method section of the papers and average brain sizes provided by Müller et al. 2018<br><br>→ ***Methods section: Literature Search, Study Selection and Data Extraction***
Studies are converted to a common reference space	YES, using the following conversion:<br><br>Coordinates reported in Talairach space were transformed into MNI space using the Lancaster transform icbm2tal implemented in GingerALE.<br><br>→ ***Methods section: Anatomical Likelihood Estimation***
Data extraction have been conducted by two investigators (ideal case) or double checked by the same investigator (state how double-checking was performed)	YES, the following authors:<br><br>**Study selection:** CS and LM (independently)<br>**Disagreements:** Solved by consensus with MM<br><br>**Data extraction:** CS and LM (independently)<br>**Disagreements:** Solved by consensus with MM<br><br>→ ***Methods section: Literature Search, Study Selection and Data Extraction***
The paper includes a table with at least the references, basic study description (e. g. for fMRI tasks: stimuli), contrasts and basic sample descriptions (e.g. size, mean age and gender distribution, specific characteristics) of the included studies, source of information (e.g. contact with authors), reference space	YES, and also the following data:<br><br>**Additional clinical sample characteristics:** Diagnosis and Duration, Abstinence Duration<br>**MRI:** method, field strength, gradient directions<br>**Pre-processing:** Smooth Kernel, Software<br>**Analysis:** Method and threshold of correcting for multiple comparisons, WM Measure, Covariates,<br>**Results:** contrasts, no. Foci, Reference Space, Source of coordinates<br><br>→ ***Table 1 and table S2***
The study protocol was previously registered and all analyses planned beforehand, including the methods and parameters used for inference, correction for multiple testing, etc.	YES:<br><br>The meta-analysis was registered before starting the search at:<br>**PROSPERO** (CRD42021231447)<br><br>→ ***Methods section***

Table S6 continued

The meta-analysis includes diagnostics	YES, the following:<br><br>fail-safe N, name and number of contributing experiments, measure, number of foci and associated contrasts regarding the revealed ALE clusters<br><br>→ ***Table 2 and table S3***
